# Association of Total Daily Physical Activity and Fragmented Physical Activity With Mortality in Older Adults

**DOI:** 10.1001/jamanetworkopen.2019.12352

**Published:** 2019-10-02

**Authors:** Amal A. Wanigatunga, Junrui Di, Vadim Zipunnikov, Jacek K. Urbanek, Pei-Lun Kuo, Eleanor M. Simonsick, Luigi Ferrucci, Jennifer A. Schrack

**Affiliations:** 1 Department of Epidemiology, Johns Hopkins Bloomberg School of Public Health, Baltimore, Maryland; 2 Center on Aging and Health, Johns Hopkins University and Medical Institutions, Baltimore, Maryland; 3 Department of Biostatistics, Johns Hopkins Bloomberg School of Public Health, Baltimore, Maryland; 4 Now with Pfizer, Cambridge, Massachusetts; 5 Division of Geriatric Medicine, Johns Hopkins University and Medical Institutions, Baltimore, Maryland; 6 Intramural Research Program, National Institute on Aging, Baltimore, Maryland

## Abstract

**Importance:**

Fragmented daily physical activity may be a sign of physiological decline that provides more powerful insight into impending mortality than total daily activity.

**Objective:**

To compare and contrast the association between total daily activity and activity fragmentation, which encompasses activity bouts and duration, and mortality risk.

**Design, Setting, and Participants:**

In this cohort study, accelerometer data from 2007 through 2015 and mortality data from 2007 through 2017 were collected from 548 adults aged 65 years and older participating in the Baltimore Longitudinal Study of Aging. The dates of analysis were November 2016 to June 2019, with data collected through December 31, 2017. Using Cox proportional hazards regression, the association between accelerometer-derived patterns of physical activity and mortality was estimated after adjusting for demographic characteristics, lifestyle factors, and comorbidities.

**Exposures:**

Minute-by-minute physical activity data were collected over a 24-hour, 7-day period (excluding times between 11:00 pm and 4:59 am) using an accelerometer. Each minute was labeled either active or sedentary, and 5 features of accelerometer data were extracted: total daily activity (defined as any activity performed throughout the day), activity fragmentation (defined as an active-to-sedentary transition probability), and 3 measures of activity bouts (<5, 5-10, and ≥10 active minutes).

**Main Outcomes and Measures:**

All-cause mortality.

**Results:**

Among 548 well-functioning older adults (mean [SD] age, 75.8 [7.2] years; 262 [47.8%] women), 61 participants (11.1%) died. Total daily physical activity was not associated with mortality risk (hazard ratio [HR], 0.90 [95% CI, 0.75-1.08]; *P* = .28). However, more fragmented physical activity patterns were associated with greater mortality risk (HR, 1.49 [95% CI, 1.02-2.19]; *P* = .04) after adjusting for age, sex, race/ethnicity, body mass index, smoking history, employment, self-reported health, grip strength, usual gait speed, comorbidities, and device wear time. In addition, more frequently engaging in activity bouts lasting less than 5 minutes was associated with greater mortality risk (HR, 1.28 [95% CI, 1.01-1.61]; *P* = .04), whereas activity bouts of 5 to 10 minutes (HR, 0.99 [95% CI, 0.58-1.69]; *P* = .97) and 10 minutes or longer (HR, 0.81 [95% CI, 0.65-1.01]; *P* = .06) were not associated with mortality risk.

**Conclusions and Relevance:**

In this cohort study of well-functioning adults aged 65 years and older, fragmented daily physical activity, particularly activity bouts lasting less than 5 minutes, was associated with greater mortality risk. These findings suggest that activity fragmentation in older adults may precede declines in functional capability and overall physical activity that typically indicate impending mortality.

## Key Points

**Question:**

Is the manner in which older adults accumulate physical activity throughout the day, beyond the total amount of physical activity, associated with their mortality risk?

**Findings:**

In this cohort study of 548 well-functioning adults aged 65 years and older, more fragmentated physical activity, but not total daily activity, was associated with increased mortality risk.

**Meaning:**

Fragmented physical activity of bouts lasting less than 5 minutes may reveal compensatory changes resulting from impaired physical function and may be associated with increased mortality risk.

## Teaser

This cohort study of adults aged 65 years and older examines whether more fragmentated physical activity, rather than total daily activity, is associated with increased mortality risk.

## Introduction

Physical activity benefits health and quality of life, particularly for adults aged 65 years and older.^1^ With aging, functional capability declines, physical activity decreases,^2^ and mortality risk increases. Previous research^3,4^ shows that volume and/or intensity measures of physical activity are associated with mortality risk, but technological advances (eg, accelerometers) present the possibility to evaluate and test whether detailed patterns of activity may be more informative to health outcomes than traditional measures of total activity, thus providing an earlier marker of future health and longevity.^5^ This is particularly important with regard to older adults, who are one of the most sedentary and rapidly growing segments of the US population.^6^

With aging and disease, activity becomes less frequent and shorter in duration, or more fragmented,^7^ as physiological capacity declines. Fragmented patterns of activity are associated with poorer physical functioning,^7,8^ a precursor of disability and frailty,^9,10^ and have been detected in cancer survivors with high fatigability and low endurance performance,^11,12^ factors consistent with accelerated aging. Thus, activity fragmentation may be a marker of a compromised physiological state and impending decline in health and functional status, making it a potential early target for intervention.

The primary objective of this study was to evaluate whether total daily physical activity and fragmentation of daily physical activity are associated with mortality among well-functioning older adults. We hypothesized that fragmentation of physical activity is more strongly associated with mortality than total daily activity. The secondary objective was to explore the duration of activity bouts, in association with mortality. We hypothesized that shorter, more fragmented bouts of activity, as opposed to longer bouts, are associated with higher mortality risk.

## Methods

### Study Design and Population

Data are from the Baltimore Longitudinal Study of Aging (BLSA), with mortality data collected between 2007 and 2017. Dates of analysis were November 2016 to June 2019. The BLSA is an ongoing study conducted by the National Institute on Aging Intramural Research Program. The BLSA’s enrollment criteria and sample details have been published previously.^13^ Briefly, BLSA enrollment requires being aged 20 years or older, with no cognitive impairment, functional limitation, or chronic disease except for hypertension or cancer diagnosis within the past 10 years. When enrolled, participants are followed for life and attend periodically scheduled comprehensive health, cognitive, and functional assessments every 1 to 4 years, depending on age. Assessments are completed over a 3-day visit to the National Institute on Aging Clinical Research Unit at Harbor Hospital in Baltimore, Maryland. Trained and certified study staff administer all evaluations according to standardized protocols. All participants provide written informed consent at each visit, and the National Institute for Environmental Health Sciences approved the study protocol. This cohort study follows the Strengthening the Reporting of Observational Studies in Epidemiology (STROBE) reporting guideline.

### Accelerometer Variables

To measure physical activity in community-dwelling settings, participants were fitted with the Actiheart monitor (CamNtech) during the last day of their BLSA clinic visit. The device, which contains a uniaxial accelerometer and a heart rate monitor, was positioned horizontally on the chest at the third intercoastal space using 2 standard electrocardiogram electrodes. Participants were instructed to wear the Actiheart monitor for 7 consecutive days. The monitor collects movement in units of gravity at a sampling rate of 32 Hz per second and preprocesses data into 1-minute epoch level activity counts (unitless quantities of movement). After the collection period, participants returned the Actiheart to the Clinical Research Unit via express mail. Data were downloaded using Actiheart software version 4.0.103 (CamNtech).

Participants with at least 3 valid days (defined as having <5% of missing data) of activity data were included in the analysis. For valid days, missing values were imputed as the mean activity counts per minute over all available days for each participant with the assumption that days with less than 5% of data missing are not substantially different from days with no missed data.^14^ Of the total accelerometer data, 2478 minutes, or 0.04% of data, were imputed. To determine active and sedentary time, each accelerometer minute was labeled as active if activity counts reached a threshold of 10 or more counts per minute or as sedentary if activity counts were fewer than 10 counts per minute.^11,14,15^ Activity bouts were defined as consecutive minutes spent in either an active or sedentary state.

Only the activity data between 5:00 am and 10:59 pm (deemed as the waking period) were considered. Three types of summary variables were created in the waking period: total active minutes, an activity fragmentation index, and total minutes spent in each of 3 different activity bout lengths (<5, 5-10, and ≥10 minutes). Total activity minutes were calculated by summing the number of active minutes and calculating the mean across wear days for each participant. Activity fragmentation was defined using the active-to-sedentary transition probability, calculated as the reciprocal of the mean activity bout length for each participant. To gain context of the patterns of bout lengths, active minutes spent in bouts of less than 5, 5 to 10, and 10 or more minutes were calculated. The shorter lengths were chosen on the basis of previous publications to represent short, medium, and long bouts of activity.^16,17^

### Mortality

Vital status was determined using telephone follow-up, correspondence, and searches of the National Death Index through December 31, 2017. Date and cause of death were adjudicated in consensus by 3 physicians who reviewed death certificates, medical records, correspondence, and other available material. For this analysis, all-cause mortality was used.

### Covariates

Age, sex, self-identified race/ethnicity, current work status, smoking history, and self-described general health were self-reported to study staff using a standardized interview questionnaire. Body mass index was calculated as the weight in kilograms divided by height in meters squared. Maximum grip strength (kilograms) was measured using a hydraulic handheld dynamometer (Jamar) from 3 trials on each hand. Usual gait speed was measured over a 6-m course, with the faster of 2 trials used for analysis. Participants self-reported whether they were ever told by a doctor or other health professional that they had any of the following conditions: cardiovascular disease, including angina, myocardial infarction, congestive heart failure, peripheral arterial disease, and vascular-related procedures; hypertension or high blood pressure; high cholesterol or triglyceride levels; stroke and transient ischemic attack; chronic bronchitis, emphysema, chronic obstructive pulmonary disease, or asthma; diabetes, glucose intolerance, or high blood glucose levels; cancer, malignant growth, or malignant tumor, not including basal or squamous cell cancers; arthritis or osteoarthritis; connective tissue disorders, including rheumatoid arthritis, gout, psoriatic arthritis, ankylosing spondylitis, lupus, ulcerative colitis, Crohn disease, scleroderma, vasculitis, or polymyositis; or kidney disease, nephritis, or renal insufficiency. Responses were summed and categorized into a morbidity index score (0, 1, and ≥2 morbid conditions).

### Statistical Analysis

Accelerometer data were collected for 888 BLSA participants between 2007 and 2015. Among them, 849 participants had at least 3 days of valid accelerometer data required for analysis (days with <5% of data missing).^14^ Those who were younger than age 65 years (284 participants), missing grip strength data (11 participants), and missing usual gait speed data (6 participants) were excluded, resulting in a final analytic sample of 548 participants. Baseline participant characteristics and accelerometer metrics were summarized across vital status using mean and SD or frequency and percentage. Time to event was accumulated starting from the first day of physical activity monitoring to date of death or December 31, 2017. Cox regression models were used to estimate the association between each continuous physical activity variable (total daily activity, activity fragmentation, and percentage of activity performed in bouts of <5, 5-10, and ≥10 minutes) and mortality. For each physical activity variable, a hazard ratio (HR) and 95% CI was calculated. The proportional hazards assumption, assessed as the interaction between physical activity variable and time, was verified for each model. A priori sensitivity tests using Cox regression were conducted to test the robustness of the Cox regression model results using nonimputed, complete case data and to evaluate the Cox regression models excluding deaths occurring within the first and second years, respectively, to examine potential reverse causality. Model 1 was adjusted for baseline age, sex, and race/ethnicity. Model 2 was adjusted for model 1 covariates and body mass index, smoking history, and current employment status. Model 3 was adjusted for Model 2 covariates and self-rated health, hand grip strength, usual gait speed, an index summary of morbid conditions, and number of activity monitor wear days. Two-tailed hypothesis testing with α = .05 was used to evaluate statistical significance. Accelerometer data were processed using R statistical software (version 3.6.0, R Project for Statistical Computing), and statistical analyses were conducted using Stata statistical software version 14 (StataCorp).

## Results

Of 548 participants in the final analytic sample, the mean (SD) age was 75.8 (7.2) years, 262 (47.8%) were women, and 72% were non-Hispanic white. Over a mean (SD) of 4.4 (2.2) years of follow-up, 61 BLSA participants (11.1%) died. At baseline, compared with participants who remained alive, the participants who died were older (mean [SD] age, 81.4 [7.5] years vs 75.1 [6.9] years), more likely to be white and not employed, less likely to report good-to-excellent health (90.2% vs 98.4%), and more likely to have a history of cardiovascular disease (27.9% vs 12.5%), stroke or transient ischemic attack (14.8% vs 7.0%), diabetes (36.1% vs 20.1%), cancer (62.3% vs 37.2%), and connective tissue disease (21.3% vs 9.5%) (Table 1). Descriptive analyses suggested that the participants who died spent less time active per day (mean [SD], 4.7 [1.5] hours per day vs 5.7 [1.7] hours per day) (Table 2) but had diurnal patterns of activity similar to those of participants who survived (Figure). However, those similarities largely appeared between participants with low activity fragmentation. Participants with high activity fragmentation exhibited diminished activity throughout the day compared with participants with low activity fragmentation, particularly among participants who died (Figure). Participants who died tended to have a higher activity fragmentation index (mean [SD], 31.1% [8.0%] vs 26.4% [6.5%]), a higher percentage of short activity bouts (mean [SD], 45.4% [12.9%] vs 37.4% [11.2%]), and a lower percentage of longer activity bouts (mean [SD], 28.5% [13.1%] vs 36.6% [13.7%]) than those who were alive at follow-up (Table 2).

**Table 1.** Participant Demographic Characteristics, by Mortality Status

Characteristic	Participants, No. (%)		*P* Value
	Alive (n = 487)	Deceased (n = 61)	
Age, mean (SD), y	75.1 (6.9)	81.4 (7.5)	<.001
Female	240 (49.3)	22 (36.1)	.05
Black	112 (22.8)	6 (9.5)	.04
Body mass index, mean (SD)^a^	27.4 (4.7)	26.2 (3.7)	.06
Employed	151 (31.0)	7 (11.5)	.002
Ever smoked	195 (40.0)	32 (52.5)	.06
Good to excellent self-reported health	479 (98.4)	55 (90.2)	<.001
Hand grip strength, mean (SD), kg	30.7 (10.6)	28.4 (8.2)	.10
Usual gait speed, mean (SD), m/s	1.13 (0.2)	0.96 (0.3)	<.001
≥2 Comorbidities	392 (80.5)	52 (85.3)	.37
Cardiovascular disease	61 (12.5)	17 (27.9)	.001
Hypertension	268 (55.0)	35 (57.4)	.73
Hypercholesterolemia or dyslipidemia	322 (66.1)	40 (65.6)	.93
Stroke or transient ischemic attack	34 (7.0)	9 (14.8)	.03
Pulmonary disease	62 (12.7)	10 (16.4)	.43
Diabetes	98 (20.1)	22 (36.1)	.005
Cancer	181 (37.2)	38 (62.3)	<.001
Osteoarthritis	294 (60.4)	39 (63.9)	.59
Connective tissue disease	46 (9.5)	13 (21.3)	.005
Kidney disease	26 (5.3)	5 (8.2)	.36
Accelerometer wear, mean (SD), d	5.9 (0.9)	6.2 (1.0)	.03

^a^ Calculated as the weight in kilograms divided by height in meters squared.

**Table 2.** Descriptive Accelerometer Metrics, by Mortality Status

Variable	Mean (SD)	
	Alive (n = 487)	Deceased (n = 61)
Total daily hours spent physically active, h/d	5.7 (1.7)	4.7 (1.5)
Fragmentation index, %^a^	26.4 (6.5)	31.1 (8.0)
Daily activity spent in bouts, %		
<5 min	37.4 (11.2)	45.4 (12.9)
5-10 min	24.5 (5.1)	24.6 (5.0)
≥10 min	36.6 (13.7)	28.5 (13.1)

^a^ Defined as the probability of transitioning from an active to a sedentary state.

**Figure.**
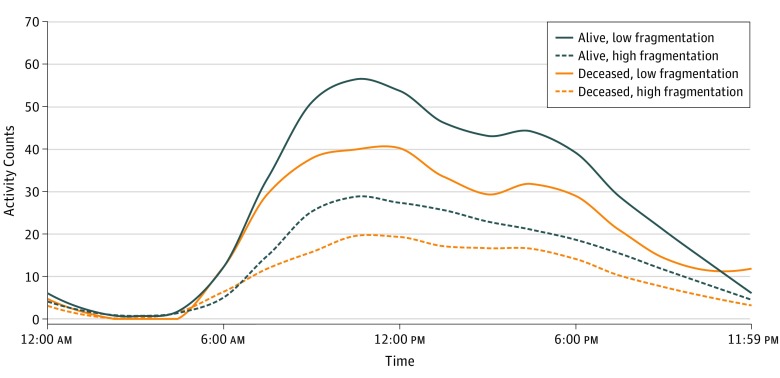
Mean Activity Patterns Stratified by Mortality and Physical Activity Fragmentation Graph depicts activity counts (unitless quantities of movement collected through accelerometers) over time. Participants who remained alive accumulated higher amounts of physical activity compared with participants who died during the course of the study. However, participants with highly fragmented physical activity had compromised diurnal activity patterns, mostly notably seen among the participants who died. Physical activity fragmentation was dichotomized at the sample median of 26%. The mean (SD) fragmentation was 21% (3%) for 255 participants who were alive and had low fragmentation, 32% (4%) for 232 participants who were alive and had high fragmentation, 23% (3%) for 19 participants who were deceased and had low fragmentation, and 35% (7%) for 42 participants who were deceased and had high fragmentation.

In fully adjusted analyses, activity fragmentation, but not total daily activity, was associated with greater mortality risk (Table 3). Specifically, a greater number of hours spent physically active each day was not associated with lower mortality risk after adjusting for age, sex, race/ethnicity, body mass index, smoking history, employment, self-reported health, grip strength, usual gait speed, comorbidities, and monitor wear time (HR, 0.90 [95% CI, 0.75-1.08]; *P* = .28). However, for each 10% higher degree of activity fragmentation, mortality risk was 49% greater (HR, 1.49 [95% CI, 1.02-2.19]; *P* = .04).

**Table 3.** Hazard Ratios for Total Activity, Activity Fragmentation, and Time Spent in Various Bout Lengths^a^

Variable	Hazard Ratio (95% CI)		
	Model 1^b^	Model 2^c^	Model 3^d^
Total physical activity, h/d	0.88 (0.74-1.03)	0.86 (0.72-1.03)	0.87 (0.73-1.03)
Activity fragmentation^e^	1.60 (1.13-2.26)	1.74 (1.19-2.54)	1.49 (1.02-2.19)
Activity spent in bouts, %^f^			
<5 min	1.35 (1.09-1.66)	1.40 (1.12-1.76)	1.28 (1.01-1.61)
5-10 min	0.89 (0.54-1.49)	0.88 (0.51-1.49)	0.99 (0.58-1.69)
≥10 min	0.78 (0.64-0.96)	0.76 (0.61-0.985	0.81 (0.65-1.01)

^a^ The sample size was 548 participants.

^b^ Adjusted for baseline age, sex, and race/ethnicity.

^c^ Model 1 plus body mass index (weight in kilograms divided by height in meters squared), smoking history, and currently working for pay.

^d^ Model 2 plus self-reported health, grip strength (kilograms), usual gait speed (meters per second), comorbidities, and activity monitor wear days.

^e^ Scaled per 10% higher degree of activity fragmentation, or a 10% higher probability of transitioning from an active to a sedentary state.

^f^ Scaled per 10% higher activity fragmentation.

When examining time spent in bouts of activity, those who spent more time in shorter rather than longer activity bouts had greater mortality risk (Table 3). For each 10% higher allocation of daily activity performed in bouts less than 5 minutes (or a >30% probability of transitioning into a sedentary state), there was a 31% greater mortality risk (HR, 1.28 [95% CI, 1.01-1.61]; *P* = .04). No association with mortality was detected between either percentage of activity spent in bouts of 5 to 10 minutes (HR, 0.99 [95% CI, 0.58-1.69]; *P* = .97) or percentage of activity spent in bouts 10 minutes or longer (HR, 0.81 [95% CI, 0.65-1.01]; *P* = .06).

Sensitivity analyses showed that the respective associations between all physical activity metrics—total activity (HR, 0.91 [95% CI, 0.76-1.09]; *P* = .30), activity fragmentation (HR, 1.48 [95% CI, 1.01-2.17]; *P* = .04), and percentage of time active in all 3 bout lengths (<5 min, HR, 1.27 [95% CI, 1.01-1.60], *P* = .04; 5-10 min, HR, 0.99 [95% CI, 0.58-1.68], *P* = .96; ≥10 min, HR, 0.82 [95% CI, 0.66-1.02], *P* = .07)—and mortality remained stable when rerunning the Cox regression models using nonimputed, complete case data (Table 4). In additionally, after excluding the only individual who died within the first year of follow-up, the association between total physical activity, fragmentation, and time spent in the various bout lengths and mortality remained the same. After excluding individuals who died within 2 years of follow-up (12 deaths), all associations remained nearly identical.

**Table 4.** Hazard Ratios for Total Activity, Activity Fragmentation, and Time Spent in Various Bout Lengths Using Nonimputed Data^a^

Variable	Hazard Ratio (95% CI)		
	Model 1^b^	Model 2^c^	Model 3^d^
Total physical activity, h/d	0.88 (0.75-1.04)	0.87 (0.73-1.04)	0.91 (0.76-1.09)
Activity fragmentation^e^	1.59 (1.12-2.24)	1.72 (1.18-2.51)	1.48 (1.01-2.17)
Activity spent in bouts, %^f^			
<5 min	1.34 (1.09-1.65)	1.39 (1.11-1.75)	1.27 (1.01-1.60)
5-10 min	0.89 (0.54-1.48)	0.87 (0.51-1.48)	0.99 (0.58-1.68)
≥10 min	0.79 (0.64-0.97)	0.77 (0.62-0.96)	0.82 (0.66-1.02)

^a^ The sample decreased from 548 to 529 participants using complete, nonimputed data. The number of participants alive decreased from 487 to 468, but the number of participants deceased remained the same at 61.

^b^ Adjusted for baseline age, sex, and race/ethnicity.

^c^ Model 1 plus body mass index (calculated as the weight in kilograms divided by height in meters squared), smoking history, and currently working for pay.

^d^ Model 2 plus self-reported health, grip strength (kilograms), usual gait speed (meters per second), comorbidities, and activity monitor wear days.

^e^ Scaled per 10% higher degree of activity fragmentation, or a 10% higher probability of transitioning from an active to a sedentary state.

^f^ Scaled per 10% higher activity fragmentation.

## Discussion

In well-functioning older adults, fragmentated daily physical activity is associated with higher mortality risk. Specifically, physical activity fragmented into higher proportions of activity bouts lasting less than 5 minutes appears to be associated with greater mortality risk. These patterns of physical activity appear to be more closely associated with mortality risk than a single measure of total volume of daily activity. Collectively, these results suggest that increasingly fragmented patterns of physical activity may be an early signal of diminished capacity that results in premature mortality. To this end, capturing fragmentation of physical activity presents a sensitive phenotypic marker of the deterioration of free-living physical activity patterns associated with mortality.

Our study shows that total daily physical activity does not explicitly indicate mortality risk in well-functioning older adults who are free of major disability. This finding appears to be contradictory to published evidence supporting an inverse association between daily physical activity and mortality in older adult populations. Although many of these studies^18,19,20,21,22^ published evidence derived from using self-reported physical activity measures, more recent studies have also detected this association using objective measures of physical activity. Manini and colleagues^23^ found that higher levels of free-living energy expenditure assessed with doubly labeled water (reference standard) was associated with a reduction in all-cause mortality in 302 high-functioning adults aged 70 to 82 years. Using an activity monitor, Ensrud and colleagues^24^ observed that less time spent in light- and moderate-intensity activities was associated with higher mortality risk in 2918 men aged 71 years and older. LaMonte and colleagues^25^ found that daily accelerometer-measured physical activity at either light or moderate-to-vigorous intensity levels was associated with lower mortality in 6382 women aged 63 to 99 years. In 3029 men and women aged 50 to 79 years old, Fishman and colleagues^3^ observed that higher volumes of total physical activity were associated with lower mortality, particularly explained by more time spent in both light and moderate-to-vigorous intensity physical activity. Although the results of our study support the magnitude and directionality of an inverse association between the amount of time spent in an active state and mortality, our findings did not achieve statistical significance. This is likely because BLSA participants were healthier and higher functioning than the general population of older adults. Together, our findings suggest that lower total physical activity among higher functioning older adults may not adequately represent increased risk of mortality.

In contrast, more fragmented daily activity appears to act as a more sensitive marker of mortality risk in healthier older adults. These results complement recent findings showing that physical activity is negatively associated with mortality, whereas sedentary time is positively associated with mortality risk.^21,26^ The concept of activity fragmentation goes beyond traditional measures of active and sedentary time by using minute-by-minute data to capture the probability of transitioning from an active to a sedentary state in free-living settings.^7,8,11^ Yet, the issue of whether deliberative or compensatory alterations in physical activity are associated with functional decline and accelerated mortality risk remains complex. Although current clinical-based functional assessments measure functional limitations,^9,27^ ceiling effects limit their ability to assess higher-order physical functioning, such as endurance capacity, fatigability, or walking efficiency.^28,29,30,31^ It is plausible that higher degrees of activity fragmentation may reflect important declines in functional capacity that limit individuals to shorter bout lengths of activity that current measurement tools cannot capture. Furthermore, activity fragmentation may capture diminished stamina, or the need to rest once active that often accompanies declining functional status,^31^ indicative of impending mortality.^27^ Additionally, previous work by our group^32^ observed that higher levels of fatigability are associated with diminished and delayed diurnal patterns of activity throughout the day and may partly explain trajectories toward premature mortality. Although these changes are largely attributable to physiological changes contributing to declines in physical function, psychological (eg, perception of the inability to climb stairs) and ecological (eg, use of elevator instead of stairs) factors contributing to activity fragmentation are possible. The current results suggest that activity fragmentation may reflect early and clinically meaningful declines in total physical activity and argue for further explorations of the underlying biological and physiological mechanisms of aging, including mitochondrial dysfunction,^33^ cellular senescence,^34^ and metabolic dysregulation.^35^

Detecting activity fragmentation may serve as an important step toward prescribing and monitoring physical activity clinically. Bayán-Bravo and colleagues^36^ showed that sedentary patterns predict greater mortality risk, whereas active patterns predict mortality risk reduction in 2851 adults aged 60 years and older. Also, Schmid and colleagues^4^ showed that a combination of high sedentary time and low physical activity was associated with short-term mortality risk in 1677 adults aged 50 years and older. Furthermore, published evidence^2,16,37,38^ shows that physical activity is typically performed in shorter rather than longer bouts of continuous activity in older adults. Our findings extend this work by providing activity fragmentation cut points that offer promising clinical utility, because fragmentation of activity into a higher number of bouts lasting less than 5 minutes, or a greater than 30% probability of transitioning into a sedentary state, may be a signal of declining functional status and impending mortality. Collectively, these results suggest that objective measures of activity fragmentation present a unique method of evaluating patterns of physical activity indicative of future health and mortality. In addition, current physical activity interventions for older adults largely focus on increasing structured physical activity at moderate or higher intensities but do little to prolong light-intensity activity engagement, particularly to reduce sedentary behavior.^17,39,40^ Reducing fragmented patterns of physical activity fits well within the World Health Organization’s 2017 Integrated Care for Older People^41^ guidelines as a novel opportunity to curb sedentary behaviors, complementary to—or possibly before—initiation of structured physical activity for the maintenance of health, intrinsic capacity, and well-being in older adults.

### Limitations

Limitations of this study include the analytic sample being largely non-Hispanic white (72%), physical activity profiles measured at 1 time point, a sample of older adults who tend to be higher functioning than the general older adult population, and low numbers of mortality events. Strengths of the study include objectively measured physical activity patterns, reduced potential residual confounding of the association between physical activity and mortality associated with recruitment of a large sample of older adults without functional decline and major disease, and the adjustment of a wide range of covariates in the analyses.

## Conclusions

In this cohort study of well-functioning adults aged 65 years and older, fragmented daily physical activity, particularly activity bouts lasting less than 5 minutes, was associated with greater mortality risk. Measures of activity fragmentation illuminate patterns of daily activity deterioration that occur with aging and disease, which potentially serve as early and sensitive markers of premature mortality. Future research is needed to assess how longitudinal changes in activity fragmentation may estimate risk of morbidity and mortality.
